# Spectral Relaxation Imaging Microscopy II: Complex Dynamics

**DOI:** 10.3390/ijms241512271

**Published:** 2023-07-31

**Authors:** Andrew H. A. Clayton

**Affiliations:** Cell Biophysics Laboratory, Department of Physics and Astronomy, Optical Sciences Centre, School of Science, Computing and Engineering Technologies, Swinburne University of Technology, Melbourne 3122, Australia; aclayton@swin.edu.au

**Keywords:** FLIM (fluorescence lifetime imaging microscopy), phasor, membranes, dynamics

## Abstract

The dynamics of condensed matter can be measured by the time-dependent Stokes shift of a suitable fluorescent probe. The time-dependent spectral correlation function is typically described by one or more spectral relaxation correlation times, which, in liquid solvents, characterize the timescales of the dipolar relaxation processes around the excited-state probe. The phasor plot provides a powerful approach to represent and analyze time and frequency-domain data acquired as images, thus providing a spatial map of spectral dynamics in a complex structure such as a living cell. Measurements of the phase and modulation at two emission wavelength channels were shown to be sufficient to extract a single excited-state lifetime and a single spectral relaxation correlation time, supplying estimates of the mean rate of excited-state depopulation and the mean rate of spectral shift. In the present contribution, two more issues were addressed. First, the provision of analytic formulae allowing extraction of the initial generalized polarization and the relaxed generalized polarization, which characterize the fluorescence spectrum of the unrelaxed state and the fully relaxed state. Second, improved methods of model discrimination and model parameter extraction for more complex spectral relaxation phenomena. The analysis workflow was illustrated with examples from the literature.

## 1. Introduction

Time-resolved spectral relaxation, whether measured in the time domain or frequency domain, is an established technique for measuring solvation dynamics in condensed phases. This method measures a time-dependent change in the spectral position of a fluorescent probe after excitation. The change in the orientational distribution of solvent molecules about the probe (i.e., the loss of orientational correlation) is recorded using a time-dependent Stokes shift (time domain at different emission wavelengths) or by a phase delay in the red edge of the emission (frequency domain).

The information obtained from spectral relaxation is the timescale for the time-dependent relaxation, which relates to the rigidity or micro-viscosity of the environment, and the equilibrium or relaxed position of the spectrum of the probe, which is related to the polarity of the environment. In biological environments such as cells, proteins, nucleic acids, membranes, and dipoles (including water) are thought to contribute to these processes.

Different approaches have been employed to extract spectral relaxation information. In one approach, time-resolved intensity decays are recorded (after pulsed excitation) at multiple emission wavelengths and are analyzed using an arbitrary sum of exponential terms. The time-resolved intensity decays are then normalized with the steady-state emission spectrum, and thence, time-resolved emission spectra are constructed. These time-resolved emission spectra can, in turn, be analyzed to provide time-dependent changes in spectral position or, alternatively, spectral width. For example, a time-dependent spectral relaxation correlation function C(t) can be defined in terms of the time-dependent normalized shift in the emission spectrum. In Equation (1), the time-dependent spectral relaxation correlation function is given in terms of the time-dependent shift in the emission peak (hν(t), in energy units) and the initial and final positions of the emission spectral peak (i.e., hν(0) and hν(∞)).

(1) $$C\left(t\right)=\frac{\left(h\nu \left(t\right)-h\nu (\infty \right))}{\left(h\nu (0\right)-h\nu \left(\infty \right))}$$

Maroncelli and Fleming [1,2] used this approach in their pioneering work on solvation in liquids. Hof’s laboratory has used this approach extensively (reviewed in [3,4,5]) to examine spectral relaxation and dipolar relaxation processes of various membrane probes embedded in phospholipid membrane vesicles. The C(t) functions are often non-single exponential and characterized by sub-nanosecond and nanosecond time constants.

For the study of spectral relaxation in living cells (i.e., with a microscope) for the purpose of imaging, photon efficiency is at a premium, and so methods using one or two emission channels (i.e., filters) have been developed. The so-called generalized polarization or GP, first described by Gratton [6,7], enables a simple characterization of the wavelength position of the emission spectrum based on measuring the normalized difference in fluorescence emission at a bluer wavelength and a red wavelength. The generalized polarization (GP) is defined by,

(2) $$\mathrm{GP}=\frac{\left(I_{B}-I_{R}\right)}{\left(I_{B}+I_{R}\right)}$$

where I_B_ is the fluorescence intensity measured in the blue channel (wavelength range defined by the experimenter), and I_R_ is the fluorescence intensity measured in the red channel (wavelength range defined by the experimenter but should be longer in wavelength than the blue channel). If intensity measurements are made as a function of time but using blue and red channels instead of a range of wavelengths, a time-dependent generalized polarization can be generated, which provides information on the timescales of spectral shifts.

(3) $$\mathrm{GP}\left(t\right)=\frac{\left(I_{B}\left(t\right)-I_{R}\left(t\right)\right)}{\left(I_{B}\left(t\right)+I_{R}\left(t\right)\right)}$$

For example, Gaus’s laboratory [8,9] made use of time-dependent generalized polarization microscopy to characterize the spectral relaxation in model membranes and in the membranes of living cells stained with membrane probes. Polarity was assessed by the excited-state lifetime population decay.

Instead of forming the GP function, another approach examines the time decay of the emission recorded in the blue part and the red part of the spectrum. Rearranging Equation (3), intensity decays can be expressed in terms of the excited state depopulation decay (I(t)) and the spectral relaxation processes (GP(t)), viz,

(4a) $$I_{B}\left(t\right)=0.5\left(\mathrm{GP}\left(t\right)+1\right)I\left(t\right)$$

(4b) $$I_{R}\left(t\right)=0.5\left(1-\mathrm{GP}\left(t\right)\right)I\left(t\right)$$

(4c) $$I\left(t\right)=(I_{B}\left(t\right)+I_{R}\left(t\right))$$

The emission measured in the blue region will be depopulated through the normal population decay of excited states but also through the shift in the emission spectrum to longer wavelengths. In contrast, the emission detected in the red will decrease due to normal population decay of excited states but show a time-dependent grow-in or increase due to the shift in emission to the red region. In the frequency domain, the extra time-dependent redshift is evidenced by an increase in the phase of the signal relative to that measured due to population decay only. By judicious selection of emission bandpass filters, Gratton’s lab [10] was essentially able to record the population decay of the excited states (I(t)) and the time decay of the intensity in the red part of the emission spectrum, thus providing evidence for spectral relaxation processes in living cells. Using the phasor approach enabled the selection of pixels with differing population decay or extent of spectral relaxation. Identification of spectral relaxation was evidenced by data points on the phasor plot, which were positioned outside of the so-called universal circle [10].

We recently demonstrated that the phasor plot (also called AB plot or polar plot) could be used for quantitative analysis of spectral relaxation phenomena from a suitable probe located in the membranes of different organelles of living cells [11]. Because the time-dependent functions in Equations (4a)–(4c) contain linked parameters, we were able to use a global analysis approach to extract relevant dynamical parameters for the simplest models of population decay and spectral relaxation. Phasors (phase and modulation data at a single modulation frequency) acquired from blue and red emission wavelengths could be analyzed to obtain a spectral relaxation time and an excited-state lifetime. Applying this approach to a Golgi-targeting membrane probe (NBD-ceramide), our analysis of probe spectral dynamics disclosed sub-nanosecond relaxation in the Golgi membranes of living cells but nanosecond relaxation in the outer plasma membranes of the cells [11]. It is noted, however, that with NBD, the precise nature of the motions giving rise to spectral relaxation is contentious, with some work indicating that probe position within the polarity gradient of the membrane may be an important factor [12].

In the present work, one of the aims is to develop a means to assess the veracity of a single-exponential spectral relaxation process. To achieve this, formulae are derived linking the observed blue and red phasors to all of the parameters in a single-exponential spectral relaxation process, including the spectral relaxation time, the excited-state lifetime, the initial generalized polarization, and the final generalized polarization. This work complements the work of Lakowicz and Balter [13] and of Weber [14]. By comparison with any of the independently measured generalized polarizations (initial, final, or steady-state), the “goodness-of-fit” of the single-exponential approach can be assessed. Simulations of complex relaxation phenomena illustrate the types of effects expected.

The other aim of the present work is to provide methods to resolve complex spectral relaxation phenomena using the phasor approach (i.e., single optical modulation frequency data). Our analysis makes use of the phasor detected in the red part of the emission. We construct a so-called red phasor ellipse plot (more precisely, a Cassini ellipse) which traces out the trajectory of all single-exponential spectral relaxations for fixed initial and final general polarizations and fixed single-exponential population decay lifetime. The parameters of a double-exponential spectral relaxation are resolved by minimizing the difference between a calculated generalized polarization and the experimental generalized polarization. We also introduce an interpretation of the motions by projection onto sub-nanosecond–super-nanosecond spectral relaxation correlation times. This model focuses on relative probabilities of different timescales of motion as opposed to attempting to resolve individual correlation times from a complex dynamical structure.

The outline of the paper is as follows. In Section 2.1, we review the theory of the red-fluorescence-edge phasor ellipse approach. In Section 2.1.1, we present formulae allowing extraction of initial and final generalized polarizations (in the context of a simple single-exponential relaxation process). In Section 2.2, we present the formulae and simulations of double-exponential spectral relaxation and show how departures from the single-exponential model can be detected through the calculation of the apparent initial and final generalized polarizations. We also describe how double-exponential relaxation processes can be resolved. In Section 2.3, we introduce IMPOSSE, which interprets motions in terms of probability amplitudes of correlation times which are pre-set over two or three orders of magnitude. In the Discussion section, we illustrate our analysis workflow with examples from the literature based on the fluorescent probes designed by Weber [15].

## 2. Results and Materials and Methods

### 2.1. Theory of the Red-Fluorescence-Edge Phasor Ellipse Approach

We will begin with a probe (with a single exponential excited-state lifetime τ) immersed in a solvent, which exhibits a single exponential-decaying spectral relaxation time (τs). We will assume emission measurements at a blue wavelength B and a red wavelength R defined by the experimenter and dependent on the probe.

After excitation with a short laser pulse, the emission spectrum will shift from its initial position (with GP(t = 0) = GP_i_) to a final relaxed emission position (GP(t = ∞) = GP_f_).

The time-dependent generalized polarization, GP(t), is,

GP(t) = (GP_i_ − GP_f_)exp(−t/τ_s_) + GP_f_ (5)

For a spectral relaxation from blue to red with increasing time, then GP_i_ > GP_f_. Uprelaxation from red to blue requires GP_f_ > GP_i_.

The decay of the excited-state population is assumed to be independent of emission wavelength and is given by the expression.

I(t) = I_o_ exp(−t/τ) (6)

If the experimenter monitors the time-dependent fluorescence at the red edge of the emission, i.e., at the red wavelength R, the observed fluorescence I_R_(t) will be a convolution of the excited state population decay with the increase in fluorescence in the red due to the time-dependent spectral shift,

I_R_(t) = 0.5I(t)(1 − GP(t)) = 0.5I_o_[(exp(−t/τ) − (GP_i_ − GP_f_)exp(−t/τ_s_) exp(−t/τ) − GP_f_ exp(−t/τ))] (7)

which can be more compactly expressed (with the substitution ϕ_s_ = (1/τ_s_ + 1/τ)^−1^

I_R_(t) = 0.5I_o_[((1 − GP_f_)exp(−t/τ) − (GP_i_ − GP_f_)exp(−t/ϕ_s_))] (8)

Equation (8) describes a double-exponential rise (negative amplitude) and decay (positive amplitude) process with the decay time τ and rise time ϕs (when GP_i_ > GP_f_ and GP_f_ < 1).

This description is essentially the same as two-state models of solvent relaxation with a Franck–Condon state or initially excited state and a relaxed state (i.e., Lakowicz and Balter [13]).

We now switch to the frequency-domain description using the phasor representation.

Defining


f_(red)_ = (1 − GP_f_)τ/[(1 − GP_f_)τ − (GP_i_ − GP_f_)ϕ_s_] (9)

We can write down the phasor components [14] corresponding to the time-resolved process from Equation (8) in terms of modulations (m) and phase shifts (θ), recorded in the wavelength region R (f = f_(red)_).

mcosθ_T_ = fmcosθ_τ_ + (1 − f)mcosθ_ϕs_ (10)

msinθ_T_ = fmsinθ_τ_ + (1 − f)msinθ_ϕs_ (11)

where

mcosθ_ϕs_ = 1/(1 + (ωϕ_s_)^2^); msinθ_ϕs_ = ωϕ_s_/(1 + (ωϕ_s_)^2^) (12)

and

mcosθ_τ_ = 1/(1 + (ωτ)^2^); msinθ_τ_ = ωτ/(1 + (ωτ)^2^) (13)

For data recorded at the blue region of the emission, the phasor components are analogous to Equations (10)–(13) except that (f = f_(blue)_).

f_(blue)_ = (1+ GP_f_)τ/[(1+ GP_f_)τ + (GP_i_ − GP_f_)ϕ_s_] (14)

Equations (1)–(14) relate a single relaxation process in the time domain to a set of phases and modulations recorded at frequency ω in the frequency domain.

#### 2.1.1. Derivation of Initial and Relaxed States for Single-Spectral-Relaxation/Single-Lifetime Model

In the circumstance that both blue and red phasors have been measured, the initial generalized polarization value and the final generalized polarization value can be determined. To achieve this, we first recall [11] that both the excited-state lifetime and the spectral relaxation time can be determined from the sine and cosine components of the fluorescence phasors recorded at the blue (mcosθ_B_, msinθ_B_) and red (mcosθ_R_, msinθ_R_) emission regions. Defining

grad = (msinθ_R_ − msinθ_B_)/(mcosθ_R_ − mcosθ_B_) and (15)

int = msinθ_R_ − (grad ∗ mcosθ_R_), (16)

the time constants are given by

τ = (1 + (1 − 4int(int + grad))^0.5^)/(2ωint) (17)

ϕ_σ_ = (1 − (1 − 4int(int + grad))^0.5^)/(2ωint) (18)

**Figure 1:**
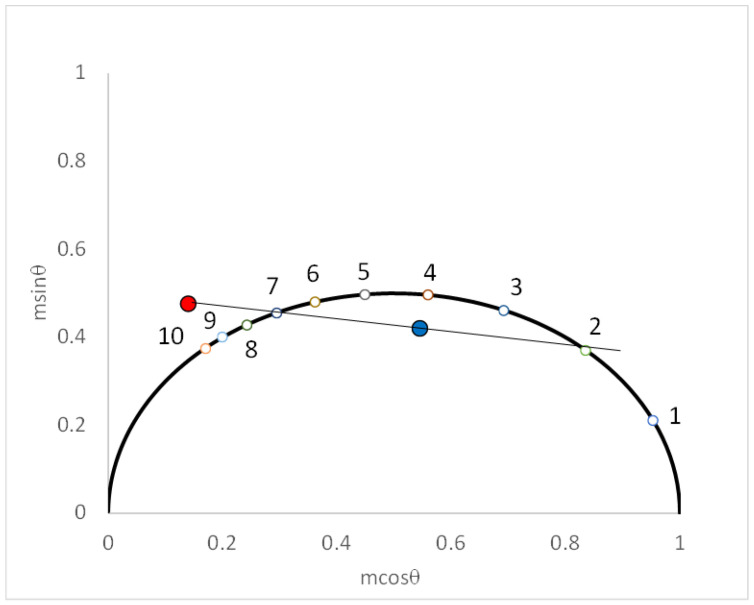
Phasor plot for single-exponential spectral relaxation and single-exponential population decay. Simulated phasor positions for blue-detected (blue symbol) and red-detected (red symbol) emission obtained from Equations (5)–(14) with parameters GP_i_ = 0.6, GP_f_ = −0.6, τ = 7 ns, τ_s_ = 3 ns, and ω = 0.2217 rad/ns. Black semi-circle denotes phasor positions of all single-exponential decay processes (numbers denote lifetimes in nanoseconds). Black line denotes linear extrapolation of the blue and red phasor points. Intersection of line with semi-circle occurs at two positions which correspond to the population decay lifetime and the joint relaxation time, respectively.

Equations (17) and (18) were derived [11] by solving for the intersection of a line with a circle (the circle has a radius of 0.5 and a center at (0.5, 0)). The universal circle (see bold curve in Figure 1) depicts the phasor positions for all single-exponential decaying functions.

Armed with the lifetime (τ), joint relaxation time (ϕ_s_), and the measured blue (mcosθ_B_, msinθ_B_) and red (mcosθ_R_, msinθ_R_) emission phasors, we can determine the GP_i_ and GP_f_,

GP_f_ = −(y − x)/(y + x) (19)

GP_i_ = (1 − x)GP_f−_x (20)

where

x = (τ/ϕ_s_)(1 − (1/F_B_); y = (τ/ϕ_s_)(1 − (1/F_R_)) (21)

and,

F_B_ = (mcosθ_B_ − mcosθ_ϕs_)/(mcosθ_τ_ − mcosθ_ϕs_); F_R_ = (mcosθ_R_ − mcosθ_ϕs_)/(mcosθ_τ_ − mcosθ_ϕs_) (22)

We have simulated the single-exponential population decay/single-exponential spectral relaxation with parameters GP_i_ = 0.6, GP_f_ = −0.6, τ = 7 ns, τ_s_ = 3 ns for an optical modulation frequency of 35 MHz (ω = 0.2217 rads/ns). Figure 1 depicts the relevant blue and red phasors on the phasor plot, together with the extrapolations to the phasor positions corresponding to the time constants in the dynamical system. Note that the line connecting the blue and red phasors intersects with the universal circle at positions corresponding to τ = 7 ns and to ϕ_s_ = 2.1 ns, from which we deduce τ_s_ = 3 ns, as expected.

We have carried out simulations of the simple model, and the results are shown in Table 1. For the simple model (single lifetime, single spectral relaxation time, GP_i_ and GP_f_), good agreement is seen between the input parameters in the model and the output parameters (as derived from the calculated phasors and Equations (1)–(22)), as expected.

These simulations suggest that provided a single-lifetime/single-spectral-relaxation-time model is appropriate, the initial and final general polarizations can be determined. Conversely, if a more complex model for spectral relaxation is suspected, an inspection of the values of the generalized polarization derived can be used to test the veracity of the single-exponential model via comparison of any of the calculated generalized polarizations with those observed experimentally. We will return to this point in the next section, where more complex models of spectral relaxation will be treated more explicitly.

### 2.2. Complex Models of Spectral Relaxation (Two Spectral Relaxation Times and One Lifetime)

The next logical model to consider is one lifetime and two spectral relaxation times. In the time domain, one can write the time-dependent generalized polarization as,

GP(t) = (GP_i_ − GP_f_) [A_1_ exp(−t/τ_s1_)+ A_2_ exp(−t/τ_s2_)] + GP_f_ (23)

where GP_i_, GP_f_ were defined previously, τ_s1_ and τ_s2_ are the two spectral relaxation times, and A_1_ and A_2_ are the associated amplitudes of the two spectral relaxation processes (NB: A_1_ + A_2_ = 1). The depopulation decay is assumed to be single exponential and given by Equation (2).

For time constants ϕ_s1_ and ϕ_s2_ with fractional fluorescence contributions β_red_ and (1 − β_red_), the composite phasor components of the emission detected in the red are given by,

mcosθ_T_ = β_red_ mcosθ_s1_ + (1 − β_red_)mcosθ_s2_ (24)

msinθ_T_ = β_red_ msinθ_s1_ + (1 − β_red_)msinθ_s2_ (25)

where mcosθ_s1_, msinθ_s1_ are from Equations (9)–(14) with ϕs = ϕ_s1_ and mcosθ_s2_, msinθ_s2_ are from Equations (9)–(14) with ϕs = ϕ_s2_

Note that the fractional fluorescence contributions are related to the amplitudes (A_1_ and A_2_) and the integrated intensity (quantum yield) detected in the red emission channel. For emission detected in the red part of the spectrum, the fractional fluorescence ratio is given by the expression,

β_red_/(1 − β_red_) = (((1 − GP_f_)τ − (GP_i_ − GP_f_)ϕ_s1_)A_1_)/(((1 − GP_f_)τ − (GP_i_ − GP_f_)ϕ_s2_) A_2_) (26)

where all parameters were defined previously.

Analogously, the phasor components for a two-component relaxation for the blue part of the emission can also be derived using Equations (24) and (25) provided the correct f_blue_ (see Equations (10)–(14)) and β_blue_ are used. The expression for β_blue_ is given in Equation (23).

β_blue_/(1 − β_blue_) = (((1 + GP_f_)τ + (GP_i_ − GP_f_)ϕ_s1_)A_1_)/(((1 + GP_f_)τ + (GP_i_ − GP_f_) ϕ_s2_) A_2_) (27)

Given the relevant excited-state and spectral shift parameters (GP_i_, GP_f_, τ, τ_s1_, τ_s2_, A_1_, and A_2_), Equations (24)–(27) allow calculation of blue and red phasors at a given optical modulation frequency, ω.

We first wish to use these equations to simulate the effect of a two-component relaxation on apparent parameters derived from the single-exponential relaxation model. To achieve this, we simulated blue and red phasors with Equations (24)–(27) and then used Equations (15)–(22) to extract single-exponential relaxation model parameters. The models simulated, and the extracted parameters obtained, are collected in Table 2.

For a two-component process with spectral relaxation times of 0.1 ns and 3 ns and an excited-state lifetime of 7 ns, it is seen that both the initial generalized polarization and the apparent single relaxation time deviate significantly from the ideal values of 0.6 and 3 ns, as the contribution from the 0.1 ns component is increased. Interestingly both the depopulation time of 7 ns and the relaxed final generalized polarization seem to be not significantly affected by the presence of the additional short relaxation component (input GP_f_ = −0.6, output GP_f_ = −0.594 to −0.598; τ input = 7 ns; τ output = 7 ns). Similar observations can be gleaned for the 1 ns and 3 ns simulations (see Table 2). However, when a long-lived relaxation of 100 ns is added to a 3 ns component, it is the initial generalized polarization value that is least perturbed (input GP_i_ = 0.6, output GP_i_ = 0.566 to 0.582), while the final generalized polarization and the apparent single relaxation time are the most perturbed parameters. These simulations serve to illustrate how departures from simple, single-exponential relaxation can impact computed generalized polarization parameters. Because generalized polarization values can be measured or estimated experimentally, the veracity of an assumed single-exponential process can be assessed. Moreover, the simulations reported in Table 2 suggest how discrepancies between measured and computed generalized polarizations may be used to provide additional information. For example, an apparent GP_i_ value (calculated with Equations (15)–(21)) which is significantly less than an expected GP_i_ value from experiments would indicate that (1) a single-exponential relaxation model is inadequate and (2) an additional short-lived relaxation (or set of relaxations) is present. Analogously, an apparent GP_f_, which is larger than expected, would also indicate the inadequacy of a single relaxation process model and further infer the need to include a longer timescale process. We next consider how to explicitly determine a two-component relaxation model. Parenthetically it is noted that the generalized polarization approach that we take here is completely analogous to our previously published approach for time-resolved fluorescence polarization anisotropy [16,17].

**Figure 2:**
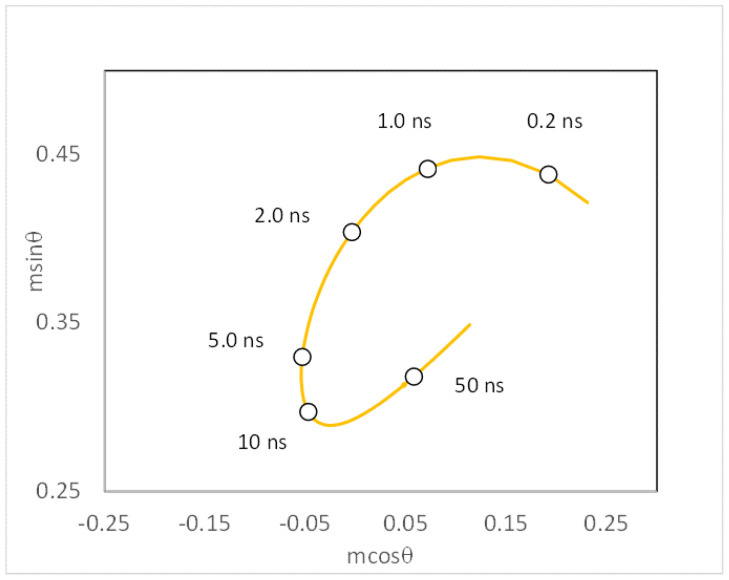
Red-edge phasor depicting the trajectory of single-exponential spectral relaxation times for fixed GP_i_ = 0.95 and GP_f_ = 0.25, τ = 3.6 ns, and ω = 0.52 rad/ns (80 MHz). Phasor positions corresponding to spectral correlation times of 0.2 ns, 1 ns, 2 ns, 5 ns, 10 ns, and 50 ns are denoted with open circles.

Given an initial fixed generalized polarization, a final fixed relaxed generalized polarization, a fixed excited-state lifetime, and a fixed frequency, one can generate a so-called red-edge phasor ellipse plot by plotting the phasor components (Equations (9)–(13)) over a range of spectral relaxation correlation times (i.e., τ_s_ = 0.01 to 1000 ns). An example of a red-edge phasor plot is in Figure 2, corresponding to parameters GP_i_ = 0.95, GP_f_ = 0.25, τ = 3.6 ns, and ω = 0.52 rad/ns (80 MHz). Note the Cassini-ellipse-like trajectory as the spectral relaxation time is varied. If the experimental data point E(mcosθ, msinθ) lies on the red-edge phasor ellipse, then a single-exponential spectral relaxation model is adequate to describe the data, and the relevant dynamical parameters can be determined by the position of the point E on the red-edge phasor ellipse. If the experimental point E lies inside the red-edge phasor ellipse plot, then a single-exponential spectral relaxation is not sufficient to describe the dynamics. One would then consider a two-component model. P(mcosθ_s1_, msinθ_s1_) and Q(mcosθ_s2_, msinθ_s2_) are the positions on the red-edge fluorescence phasor ellipse plot corresponding to the time constants ϕ_s1_ and ϕ_s2_. By vector algebra, these two phasor positions and the red-edge experimental phasor must lie on the same line (Equations (24) and (25)).

To resolve the two-component processes, I introduce a third coordinate axis in the Z-direction of the red-edge phasor ellipse plot, which contains information about the steady-state generalized polarization.

For a given τ_s_ and fixed τ, GP_i_, GP_f_, and ω, the steady-state GP is given by the expression,

GP = (GP_i_ − GP_f_)/(1 + (τ/τ_s_)) + GP_f_ (28)

Thus, for τ_s1_ and τ_s2_,

GP(τ_s1_) = (GP_i_ − GP_f_)/(1 + (τ/τ_s1_)) + GP_f_ (29)

GP(τ_s2_) = (GP_i_ − GP_f_)/(1 + (τ/τ_s2_)) + GP_f_ (30)

The observed GP for a mixture of two relaxation processes is the weighted sum of the GPs corresponding to the two relaxations,

GP(sum) = A_1_ GP(τ_s1_) + A_2_ GP(τ_s2_) (31)

Given the experimental red phasor and steady-state GP values, the problem at hand is to minimize the difference between the theoretical GP (Equation (31)) and the experimental GP. This is performed by first guessing a value for one of the relaxation times, thence finding the second relaxation time and associated amplitudes (by vector algebra) and GP (theory). This process is repeated until the minimum variance between experimental GP and theoretical GP is reached. These equations can be solved numerically or graphically, as we have shown previously for analogous problems of this type [16,17].

To provide an example of the approach, we have simulated a double-exponential spectral relaxation (parameters: GP_i_, GP_f_, A_1_, A_2_, τ_s1_, τ_s2_) = (0.6, −0.6, 0.25, 0.75, 0.9 ns, 9 ns), excited-state lifetime of 7 ns, and optical modulation frequency of 0.25 rad/ns (or 40 MHz) and then tried to solve the inverse problem by extracting the two spectral relaxation correlation times. Table 3 lists the initial guesses for τ_s1_ (and therefore A_1_, A_2_, and τ_s2_ by vector algebra) and the calculated GP. It is clear from Table 3 that the correct spectral relaxation correlation times can be recovered. We can also gain an estimate of our confidence in the extracted correlation times by setting an error threshold for GP. For example, if we allow an error of 1% in GP, then the error in the parameters A_1_, A_2_, τ_s1_, τ_s2_ are ±0.002, 0.002, 0.02 ns, 0.4 ns.

A geometrical approach to the problem can be visualized as follows. A 3D plot of the red-edge phasor ellipse with the GP values as the z-value resembles something like a half turn of a spiral staircase. Given a vertical pole located inside the spiral staircase at point E(mcosθ, msinθ) with height (z = GP), the problem at hand is then to find the location of a thin beam that touches the top of the vertical pole and touches the staircase at two points. The intersection of the beam with the two points on the spiral staircase corresponds to the two relaxation times, while the positions of these points relative to the experimental point are related to the fractional contribution β, as per vector algebra.

### 2.3. Interpretation of Motions by Projection onto Sub-Nanosecond–Super-Nanosecond Spectral Relaxation Correlation Times (IMPOSSE)

Membranes in living cells are complex entities, and it is unlikely that this complexity can be captured by models involving one or two spectral relaxation correlation times. Indeed, high-resolution time-resolved spectroscopy measurements in pure lipid membranes reveal dynamics that can span at least two orders of magnitude. Instead of focusing on fitting individual correlation times, we propose a model analogous to that used in NMR, which allows for an array of correlation times that are parametrized to be equally spaced on a logarithmic scale [18]. The model then focuses on the probability amplitudes of motions occurring on different timescales as opposed to extracting individual correlation times.

For the model at hand, we define a parametric time constant τ_c_.

The i-th time constant is given by the expression

τ_i_ = α^i−1^ τ_c_ (32)

where

α = (τ_max_/τ_c_)^1/n−1^ (33)

For experiments conducted with a single optical modulation frequency considered here, n = 3. The range of correlation time magnitudes can be limited to either two or three orders of magnitude. For two orders of magnitude α = 10 (and the three correlation times are τ_c_, 10 τ_c_, and 100 τ_c_) and for three orders of magnitude α = 31.62 (and the three correlation times are τ_c_, 31.62 τ_c_, and 1000 τ_c_).

Given the experimental phasor of the red-edge fluorescence and the steady-state GP value, τ_c_ is adjusted until the theoretical GP value matches the experimental GP value. The amplitudes associated with each scale of motion can be easily extracted using standard vector algebra (i.e., by extension from Equations (24)–(26)).

It is stressed that this approach and model are not intended to replace high-resolution time-resolved fluorescence spectroscopy experiments but instead should be viewed as a representation based on timescales only, which gives the relative probability density of being sub-nanosecond, nanosecond, or super-nanosecond.

## 3. Discussion

Perhaps the best way to evaluate the models proposed here is to provide an example from the literature. The intent is to illustrate model discrimination and to show how the phasor approach and the time-dependent generalized polarization are complementary.

**Figure 3:**
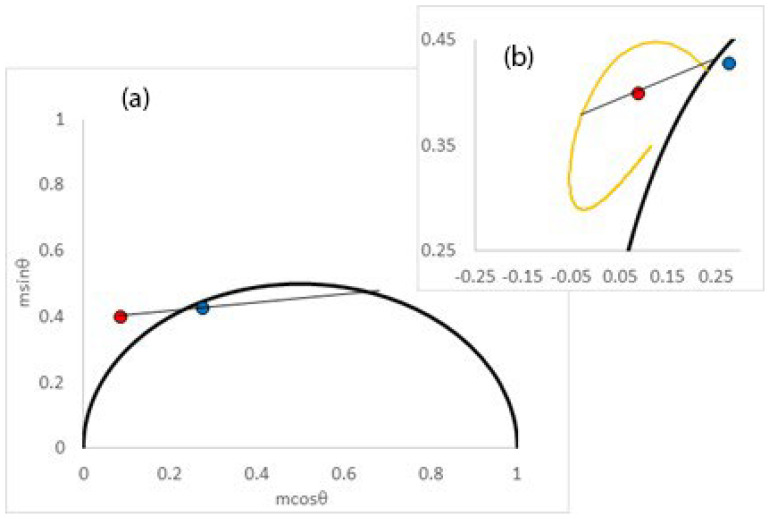
Representative phasor diagrams of lifetime decay and spectral relaxation for Laurdan in the internal membranes of living cells. Blue circle denotes phasor position using blue filter, and red circle denotes phasor position collected using green filter. (**a**) Single-exponential spectral relaxation model. Black line indicates linear extrapolation of the blue and red phasor points. Intersection of line with semi-circle occurs at two positions which correspond to the population decay lifetime and the joint relaxation time, respectively. (**b**) Inset: double-exponential spectral relaxation decay model. Orange curve depicts red-edge phasor ellipse plot, which traces out all single-exponentially decaying spectral relaxations for GP_i_ = 0.95, GP_f_ = 0.25, and τ = 3.6 ns. Red circle denotes position of a representative phasor only. Note the position of the red phasor inside the ellipse implies non-single-exponential spectral relaxation. Black line denotes interpretation in terms of a double-exponential spectral relaxation decay model. The intersection of the line with the ellipse denotes the two spectral relaxation times. Reprinted/adapted with permission from Ref. [10], 2013, Elsevier.

**Figure 4:**
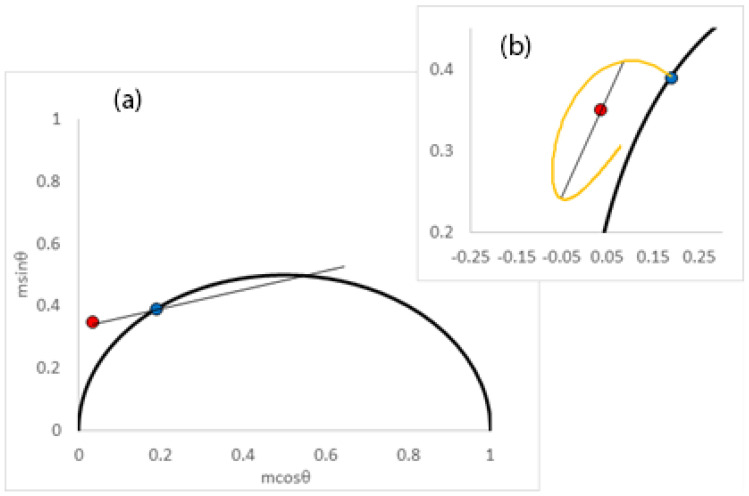
Representative phasor diagrams of lifetime decay and spectral relaxation for Laurdan in the plasma membranes of living cells. Blue circle denotes phasor position using blue filter, and red circle denotes phasor position collected using green filter. (**a**) Single-exponential spectral relaxation model. Black line indicates linear extrapolation of the blue and red phasor points. Intersection of line with semi-circle occurs at two positions which correspond to the population decay lifetime and the joint relaxation time, respectively. (**b**) Inset: double-exponential spectral relaxation decay model. Orange curve depicts red-edge phasor ellipse plot, which traces out all single-exponentially decaying spectral relaxations for GP_i_ = 0.95, GP_f_ = 0.25, and τ = 4.1 ns. Red circle denotes position of a representative phasor only. Note the position of the red phasor inside the ellipse implies non-single-exponential spectral relaxation. Black line denotes interpretation in terms of a double-exponential spectral relaxation decay model. The intersection of the line with the ellipse denotes the two spectral relaxation times. Reprinted/adapted with permission from Ref. [10], 2013, Elsevier.

Laurdan is a widely used membrane probe, originally developed by Gregorio Weber for examining dipolar relaxation. It provides a nice example of the formalism outlined here because Gaus’s lab [9] has shown that the time-dependent generalized polarizations from this probe are double exponential in cell membranes, while Gratton’s lab [10] has published extensively with this probe to reveal spatial heterogeneity with the phasor plot. The formalism developed here allows data from the phasor plot to be analyzed in terms of a double-exponential generalized polarization decay model. To provide a concrete example of how to implement the models developed herein, we choose values of blue and red phasors to correspond closely to those reported by Gratton’s lab [10] for the Laurdan probe in living cells (note that blue phasor refers to data collected with 460/80 nm filter and red refers to data collected with 540/50 nm filter [10]). Representative phasor components reported from the interior membranes and plasma membranes are collected in Table 4 and shown in Figure 3 (interior membranes) and Figure 4 (plasma membranes). Before proceeding to analyze data, we need values of the expected initial and final generalized polarizations (GP_i_ and GP_f_) for Laurdan. Based on the published spectrum of the chromophore in Laurdan (i.e., Prodan) in hydrocarbon solvent [15], we estimate GP_i_ = 0.95. An estimate of GP_f_ can be obtained from the emission spectrum of Laurdan in a fast-relaxing environment of polarity equivalent to the interface of a membrane. The estimated GP for Laurdan in ethanol (based on the spectrum of Prodan in ethanol) was 0.27. We therefore take a value of GP_f_ = 0.25.

Turning first to the data from the internal membranes (Figure 3), we note that the phase of the red-detected emission is larger than the total detected emission, as reported by Gratton [10]. Secondly, the red-detected phasor point lies outside the universal circle. Both of these observations imply an excited state process, such as spectral relaxation. In the context of a simple single excited-state lifetime, single relaxation process model, we could extract the lifetime and spectral relaxation correlation time using Equations (15)–(18) and found values of 3.6 ns and 2.55 ns, an apparent GP_i_ = 0.76, and a GP_f_ = 0.35 (using Equations (15)–(22)). Figure 3a contains the red- and blue-detected phasors along with the linear fit to the simple single-exponential relaxation model. The discrepancy between the apparent initial GP value (GP_i_ = 0.76) and the expected value GP_i_ = 0.95 implies that a more complex model than a simple single relaxation model is needed. Moreover, the calculated steady-state GP value with the simple model (GP = 0.52) was different from the average steady-state GP value reported from the interior membranes (GP = 0.43) [10]. The blue-detected phasor is not really a blue-edge phasor but rather is more representative of the population decay of the excited state because it was acquired with a broadband filter [10]. We therefore sought another way to analyze the data with a single-exponential relaxation process but using only the red-edge-detected phasor. Another approach was to fix the depopulation decay time to 3.6 ns, fix the relaxed GP_f_ value to 0.25, and then adjust GP_i_ until the red-detected phasor lies on the red-phasor ellipse. This analysis yielded similar GP_i_ and τ_s_ values to when both phasors were employed. Thus GP_i_ = 0.73, and τ_s_ = 2.2 ns with this model (c/f GP_i_ = 0.76 and τ_s_ = 2.55 ns with the previous model). Again, the small value of GP_i_ as compared to the expected value indicates that another spectral relaxation process is present, likely to be on the sub-nanosecond timescale. Another way of seeing the inadequacy of a single-exponential relaxation model is by comparing the position of the red-edge-detected experimental phasor point relative to the red-edge phasor ellipse plot (plot constructed with GP_i_ = 0.95, GP_f_ = 0.25, and τ = 3.6 ns). As can be seen in Figure 3b, the red-edge phasor lies well inside the red-edge phasor ellipse plot, which indicates a non-single-exponential process is responsible for the spectral relaxation. Now turning to the two-component relaxation model, the dipolar relaxation in the internal membranes was characterized by a major component with a relaxation time of 2.7 ns (amplitude fraction = 0.66) and a minor sub-nanosecond component (0.03 ns with amplitude fraction = 0.34). The amplitude-weighted average spectral relaxation time calculated from these results was 1.8 ns. The IMPOSSE analysis with two orders of magnitude time range disclosed amplitudes (0.33, 0.01, 0.66) associated with 0.027, 0.27, and 2.7 ns timescales, respectively. The model essentially reduced to the two-correlation time model (the amplitude associated with the 0.27 ns motion was negligible). IMPOSSE analysis with three orders of magnitude failed to return a good fit or physically reasonable values. The values of the correlation times can be compared with time-resolved spectral shift measurements of Laurdan in model membranes. Thus, Amaro et al. [4] reported sub-nanosecond dynamics (0.3 ns) from Laurdan in POPC membranes at 37 °C (in the liquid-disordered phase), while nanosecond timescale spectral dynamics (2.7 ns) from POPC membranes containing 10% cholesterol at 23 °C (in the more ordered phase). Because inner membranes in the endoplasmic reticulum and the Golgi tend to have lower cholesterol and are more loosely packed (than plasma membranes), the timescales of the spectral dynamics detected here appear to be physically reasonable.

The analysis of the data relevant to the plasma membrane produced results that were distinctly different. The single relaxation time model yielded dynamic parameters consistent with a longer depopulation decay (τ = 4.1 ns) and longer spectral relaxation time (τ_s_ = 2.7 ns) and GP values (GP_i_ = 0.96; GP_f_ = 0.88) suggestive of a more restricted relaxation process than in the interior membranes. Figure 4a depicts the phasor positions together with the linear extrapolation needed to extract the excited-state lifetime and spectral relaxation time. The large value of GP_f_ = 0.88 indicates the presence of an additional, longer relaxation process. The calculated steady-state value for the GP from this model was 0.91 is also larger than the experimental average of 0.6. The large discrepancy between the model-derived GP value and the experimentally determined GP value indicates that a more complex model needs to be considered for this system.

An alternative analysis for a single exponential relaxation process using only the red-detected phasor yielded parameters that were also incompatible with the expected GP_i_ (GP_i_ = 0.76 and τ_s_ = 2.8 ns). Turning to the two-component model with fixed parameters (GP_i_ = 0.95; GP_f_ = 0.25, lifetime = 4.1 ns) and a target average GP value of 0.6, we extracted two relaxation times with values of 13 ns (fraction amplitude = 0.59) and 0.7 ns (fraction amplitude 0.41). The amplitude-weighted average spectral relaxation time calculated from these results was 7.9 ns. Figure 4b depicts the phasor positions, the red-edge phasor ellipse, together with the extrapolations required to determine the two spectral relaxation times.

The IMPOSSE analysis with two orders of magnitude time range disclosed amplitudes (0.32, 0.37, 0.30) associated with 0.45, 4.5, and 45 ns timescales, respectively. Attempts to fit the IMPOSSE analysis with three orders of magnitude time range produced non-physical results.

The analysis presented here reveals that a single exponential decay process is not an adequate model to describe Laurdan spectral relaxation in membranes of living cells. Given the complexity of membranes (transverse polarity/dynamics gradient, lateral organization, chemical, and phase heterogeneities), this conclusion is perhaps not surprising. What is interesting is that the two correlation time analysis and the IMPOSSE analysis both revealed a long relaxation behavior on the timescale of tens of nanoseconds in the plasma membrane (but not detected in the inner membranes), which is probably too long to be due to dipolar relaxation of water molecules but may be due to other processes such as probe (translational and/rotational motion in the membrane [19] and/or relaxation of other species in the membrane. It is notable that Gaus’s laboratory also reported slow spectral relaxation processes in the plasma membrane using the time-resolved GP approach [9].

More work is needed to investigate this aspect further.

## 4. Conclusions

The theoretical framework for analyzing phasor-FLIM data in terms of time-dependent spectral relaxation was presented and extended to include complex dynamics. The analysis uses data acquired at a steady state to constrain possible models. Applying the formalism to literature data, we were able to show the extraction of sub-nanosecond to super-nanosecond timescale relaxations in membranes of living cells.

## Figures and Tables

**Table 1:** Simulated spectral relaxation and population decay. Note the good agreement between input model parameters and output extracted parameters.

Model	Input τ_s_	Input GP_i_	Input GP_f_	Output Parameters
1	0.1	0.6	−0.6	0.1, 0.6, −0.6
2	1	0.0	−0.6	1, 0.0, −0.6
3	3	−0.2	−0.6	3, −0.2, −0.6
4	10	0.3	−0.3	10, 0.3, −0.3

**Table 2:** Simulated spectral relaxation and population decay for various two-component spectral relaxation models (INPUT). OUTPUT reveals the fits of the two-component models to an effective single-exponential spectral relaxation decay model using theory and equations in the main text. Population decay excited-state lifetime was fixed to 7 ns in these simulations (40 MHz optical modulation frequency).

Input GP_i_; GP_f_	Input τ_1;_ τ_2_	Input A_1_; A_2_	Ouput GP_i_; GP_f_	Output τ_s_
0.6; −0.6	0.1; 3	0.2; 0.8	0.385; −0.598	2.91
0.6; −0.6	0.1; 3	0.4; 0.6	0.172; −0.596	2.78
0.6; −0.6	0.1; 3	0.6; 0.4	−0.05; −0.594	2.56
0.6; −0.6	0.1; 3	0.8; 0.2	−0.25; −0.594	2.06
0.6; −0.6	1; 3	0.2; 0.8	0.536; −0.594	2.67
0.6; −0.6	1; 3	0.4; 0.6	0.484; −0.590	2.31
0.6; −0.6	1; 3	0.6; 0.4	0.453; −0.590	1.91
0.6; −0.6	1; 3	0.8; 0.2	0.467; −0.593	1.48
0.6; −0.6	100; 3	0.2; 0.8	0.582; −0.397	3.22
0.6; −0.6	100; 3	0.4; 0.6	0.568; −0.195	3.61
0.6; −0.6	100; 3	0.6; 0.4	0.561; 0.003	4.35
0.6; −0.6	100; 3	0.8; 0.2	0.566; 0.185	6.53

**Table 3:** Trial guesses for a simulated double-exponential spectral relaxation process. The ground-truth model is highlighted in bold font.

Guess τ_s1_	τ_s2_	A1/A2	Calculated GP	True GP
0.50	3.49	0.55/0.45	−0.378	−0.329
0.80	6.25	0.72/0.28	−0.352	−0.329
0.89	8.62	0.748/0.252	−0.332	−0.329
**0.90**	**9.00**	**0.750/0.250**	**−0.329**	**−0.329**
0.91	9.41	0.752/0.248	−0.325	−0.329
1.0	15.27	0.75/0.25	−0.282	−0.329
1.2	47.83	0.635/0.365	−0.106	−0.329

**Table 4:** Representative phasor components from inner membranes and plasma membranes and a comparison of different spectral relaxation models. Note the (f) denotes parameters that were fixed in the analysis. The phasor components were chosen to correspond closely to those reported in Reference [10] (Reprinted/adapted with permission from Ref. [10], 2013, Elsevier.)

	Phasor	Phasor	Model	GP_i_	GP_f_	τ_s1_	τ_s2_	A_1_	τ
interior	(0.276, 0.428)	(0.087, 0.4)	single	0.76	0.35	2.55	0	0	3.63
interior		(0.087, 0.4)	single	0.73	0.25(f)	2.20	0	0	3.63(f)
interior		(0.087, 0.4)	double	0.95(f)	0.25(f)	2.70	0.03	0.66	3.63(f)
plasma	(0.190, 0.389)	(0.035, 0.350)	single	0.960	0.880	2.70	0	0	4.19
plasma		(0.035, 0.350)	single	0.76	0.25(f)	2.80	0	0	4.19(f)
plasma		(0.035, 0.350)	double	0.95(f)	0.25(f)	13	0.65	0.59	4.19(f)

## Data Availability

Not applicable.
